# Supplementary material for: Association between pregnancy planning or intention and early child development: A systematic scoping review
Source: PLOS Glob Public Health. 2023 Dec 5;3(12):e0002636. doi: 10.1371/journal.pgph.0002636 (PMC10697520; doi:10.1371/journal.pgph.0002636)
Supplement: S1 Appendix — (DOCX) [file pgph.0002636.s001.docx]

# Studies identified a priori

Saleem, H. T., & Surkan, P. J. (2014). Parental pregnancy wantedness and child social-emotional development. *Maternal and Child Health Journal*, *18*(4), 930–938. https://doi.org/10.1007/s10995-013-1320-z

Baydar, N. (1995). Consequences for children of their birth planning status. *Family Planning Perspectives*, *27*(6), 228–234. https://doi.org/10.2307/2136174

Crissey, S. R. (2005). Effect of pregnancy intention on child well-being and development: Combining retrospective reports of attitude and contraceptive use. *Population Research and Policy Review*, *24*(6), 593–615. https://doi.org/10.1007/s11113-005-5734-1

Jang, M., Molino, A. R., Ribeiro, M. V., Mariano, M., Martins, S. S., Caetano, S. C., & Surkan, P. J. (2021). Maternal Pregnancy Intention and Developmental Outcomes in Brazilian Preschool-Aged Children. *Journal of Developmental and Behavioral Pediatrics*, *42*(9), E15–E23. https://doi.org/10.1097/DBP.0000000000000951

de La Rochebrochard, E., & Joshi, H. (2013). Children born after unplanned pregnancies and cognitive development at 3 years: social differentials in the United Kingdom Millennium Cohort. *American Journal of Epidemiology*, *178*(6), 910–920. https://doi.org/10.1093/aje/kwt063

Hummer, R. A., Hack, K. A., & Raley, R. K. (2004). Retrospective Reports of Pregnancy Wantedness and Child Well-Being in the United States. *Journal of Family Issues*, *25*(3), 404–428. https://doi.org/10.1177/0192513X03257712

Foster, D. G., Raifman, S. E., Gipson, J. D., Rocca, C. H., & Biggs, M. A. (2019). Effects of Carrying an Unwanted Pregnancy to Term on Women’s Existing Children. *Journal of Pediatrics*, *205*, 183-189.e1. https://doi.org/10.1016/j.jpeds.2018.09.026

Carson, C., Kelly, Y., Kurinczuk, J. J., Sacker, A., Redshaw, M., & Quigley, M. A. (2011). Effect of pregnancy planning and fertility treatment on cognitive outcomes in children at ages 3 and 5: Longitudinal cohort study. *BMJ (Online)*, *343*(7820), 1–9. https://doi.org/10.1136/bmj.d4473

Carson, C., Redshaw, M., Sacker, A., Kelly, Y., Kurinczuk, J. J., & Quigley, M. A. (2013). Effects of pregnancy planning, fertility, and assisted reproductive treatment on child behavioral problems at 5 and 7 years: Evidence from the Millennium Cohort Study. *Fertility and Sterility*, *99*(2), 456–463. https://doi.org/10.1016/j.fertnstert.2012.10.029

Joyce, T. J., Kaestner, R., & Korenman, S. (2000). The effect of pregnancy intention on child development. *Demography*, *37*(1), 83–94. https://doi.org/10.2307/2648098

# Litsearchr specifications and results

We extracted all keywords (min_n=1), all words from a title that repeated at least once and separated by two stopwords (min_freq=2, min_n=1). These terms were then” pasted” to the abstract of each reference. Every term repeated at least twice was subtracted and included in the strength analysis.

term strength rank

1 unwanted pregnancy 24 1

2 pregnancy intention 26 2

3 intention 26 2

4 united 32 4

5 cognitive 34 5

6 cohort study 36 6

7 millennium 36 6

8 millennium cohort 36 6

9 planning 39 9

10 effects 40 10

11 cohort 42 11

12 outcomes 49 12

13 years 54 13

14 effect 63 14

15 study 76 15

16 development 78 16

17 children 80 17

18 pregnancy 84 18

19 child 89 19

# Search terms

|  | **Criteria** | **MESH terms** | **Litsearchr terms** | **Combined search** |
| --- | --- | --- | --- | --- |
| *Population* | Children aged 36 to 59 months old; | "Child, Preschool"[Mesh] | Child, children | ("Child, Preschool"[Mesh] OR child OR children) |
| *Exposure* | Unwanted, Unplanned, Mistimed, or Unintended pregnancy. | (("Pregnancy, Unwanted"[Mesh]) OR "Child, Unwanted"[Mesh]) OR "Pregnancy, Unplanned"[Mesh] | (Pregnancy intention) OR (unwanted pregnancy) OR (pregnancy planning) OR (pregnancy wantedness) ~~OR fertility~~ | ((("Pregnancy, Unwanted"[Mesh]) OR "Child, Unwanted"[Mesh]) OR "Pregnancy, Unplanned"[Mesh]) OR ((Pregnancy intention) OR (unwanted pregnancy) OR (pregnancy planning) OR (pregnancy wantedness)) |
| *Comparator* | Children aged 36 to 59 months old born from planned/wanted pregnancies |  |  |  |
| *Outcome* | Child development | "Child Development"[Mesh] OR "Developmental Disabilities"[Mesh] OR "Child Development Disorders, Pervasive"[Mesh] | (Child well-being) OR (cognitive AND development) | (("Child Development"[Mesh] OR "Developmental Disabilities"[Mesh] OR "Child Development Disorders, Pervasive"[Mesh]) OR (child well-being) OR (cognitive AND development)) or development*[Title] |

Excluded terms from litsearchr that did not belong to any particular criteria: cohort, cohort study, effect, fertility, millennium, millennium cohort, outcomes, reports, retrospective, study, treatment, united, well-being, years.

# PUBMED search strategy

("child, preschool"[MeSH Terms] OR ("Child"[Title/Abstract] OR "children"[Title/Abstract])) AND ("pregnancy intention"[Title/Abstract] OR "unwanted pregnancy"[Title/Abstract] OR "pregnancy planning"[Title/Abstract] OR "pregnancy wantedness"[Title/Abstract] OR ("pregnancy, unwanted"[MeSH Terms] OR "child, unwanted"[MeSH Terms] OR "pregnancy, unplanned"[MeSH Terms])) AND ("Child Development"[MeSH Terms] OR "Developmental Disabilities"[MeSH Terms] OR "child development disorders, pervasive"[MeSH Terms] OR ("child well being"[Title/Abstract] OR ("cognitive"[Title/Abstract] AND "development"[Title/Abstract] OR development*[Title])))

# OVID (Medline and EMBASE)

Database: Embase <1974 to 2022 April 18>, Ovid MEDLINE(R) and Epub Ahead of Print, In-Process, In-Data-Review & Other Non-Indexed Citations, Daily and Versions <1946 to April 18, 2022>

Search Strategy:

--------------------------------------------------------------------------------

1 exp Child, Preschool/ (1563757)

2 (child or children).ti,ab,cl,oa,kw,kf. (3076417)

3 exp Pregnancy, Unwanted/ (6231)

4 exp Pregnancy, Unplanned/ (8841)

5 exp Child, Unwanted/ (334)

6 (Pregnancy intention or unwanted pregnancy or pregnancy planning or pregnancy wantedness).ti,ab,cl,oa,kw,kf. (6681)

7 1 or 2 (3828187)

8 3 or 4 or 5 or 6 (18969)

9 7 and 8 (3417)

10 exp Child Development/ or exp Developmental Disabilities/ (182541)

11 (Child well-being or (cognitive and development)).mp. [mp=ti, ab, hw, tn, ot, dm, mf, dv, kf, fx, dq, nm, ox, px, rx, ui, sy] (177313)

12 Development*.ti. (1206378)

13 10 or 11 or 12 (1467580)

14 9 and 13 (186)

15 remove duplicates from 14 (146)

# Scopus

( TITLE-ABS-KEY ( child OR children OR preschool ) AND TITLE-ABS-KEY ( "unwanted pregnancy" OR "unplanned pregnancy" OR "pregnancy planning" OR "pregnancy intention" OR "pregnancy handedness" OR "unwanted child" ) AND TITLE-ABS-KEY ( "child development" OR "developmental disabilities" OR "child well being" OR ( cognitive AND development ) ) AND TITLE ( development* ) )

Search Sensitivity

9/10 (90%)

## Missing article(s)

Carson, C., Redshaw, M., Sacker, A., Kelly, Y., Kurinczuk, J. J., & Quigley, M. A. (2013). Effects of pregnancy planning, fertility, and assisted reproductive treatment on child behavioral problems at 5 and 7 years: Evidence from the Millennium Cohort Study. *Fertility and Sterility*, *99*(2), 456–463. <https://doi.org/10.1016/j.fertnstert.2012.10.029>

## Discussion

It seems the authors tried to differentiate this second article of the study by using “child behaviour” as an outcome instead of child development. Throughout the manuscript sections and metadata, “development” is omitted and replaced by this term. The keywords for the article were ‘Pregnancy planning,’ ‘ART,’ ‘IVF,’ ‘Strengths and Difficulties Questionnaire,’ and ‘behavior.’ If we consider forward and backward citation chasing for all articles included in the final sample, then the sensitivity is 100%. However, we desired to report a conservative estimate.
