# Supplementary material for: Association between pregnancy planning or intention and early child development: A systematic scoping review
Source: PLOS Glob Public Health. 2023 Dec 5;3(12):e0002636. doi: 10.1371/journal.pgph.0002636 (PMC10697520; doi:10.1371/journal.pgph.0002636)
Supplement: S2 Appendix — (DOCX) [file pgph.0002636.s002.docx]

# List of excluded reports organized by reason

## Wrong population

### Children younger than 36 months

C., McCrory, and McNally S. 2013. “The Effect of Pregnancy Intention on Maternal Prenatal Behaviours and Parent and Child Health: Results of an Irish Cohort Study.” *Paediatric and Perinatal Epidemiology* 27 (2): 208–15.

### Children older than 60 months

#### Czech cohort(s)

Z., Dytrych, Matejcek Z., Schuller V., David H.P., and Friedman H.L. 1975. “Children Born to Women Denied Abortion.” *Family Planning Perspectives* 7 (4): 165–71.

Z., Matejcek, Dytrych Z., and Schuller V. 1975. “A Prague study of children born from unwanted pregnancies. II. Girls and boys (Czech).” *Psychologia a Patopsychologia Dietata* 10 (4): 291–306.

Z., Matejcek, Dytrych Z., and Schuller V. 1978. “Children from Unwanted Pregnancies.” *Acta Psychiatrica Scandinavica* 57 (1): 67–90.

HP, David, and Z Matĕjcek. 1981. “Children Born to Women Denied Abortion: An Update.” *Family Planning Perspectives* 13 (1): 32–34. <https://pubmed.ncbi.nlm.nih.gov/7215518/>.

Z., Matejcek, Dytrych Z., and Schuller V. 1985. “Follow-up Study of Children Born to Women Denied Abortion.” *Ciba Foundation Symposium* 115: 136–49.

Z., Matejcek, Dytrych Z., and Schuller V. 1986. “Children born from unwanted pregnancy during early adolescence.” *Demografie*, Deti narozene z nechteneho tehotenstvi, 28 (4): 313–22.

L., Kubieka, Matejeck Z., David H.P., Dytrych Z., Miller W.B., and Roth Z. 1995. “Children from Unwanted Pregnancies in Prague, Czech Republic Revisited at Age Thirty.” *Acta Psychiatrica Scandinavica* 91 (6): 361–69.

David, Henry P. 2006. “Born Unwanted, 35 Years Later: The Prague Study.” *Reproductive Health Matters* 14 (27): 181–90.

David, Henry P. 2011. “Born Unwanted: Mental Health Costs and Consequences.” *American Journal of Orthopsychiatry* 81 (2): 184–92.

#### Danish cohort(s)

B., Bay, Mortensen E.L., Golombok S., Hohwu L., Obel C., Henriksen T.B., and Kesmodel U.S. 2016. “Long-Awaited Pregnancy: Intelligence and Academic Performance in Offspring of Infertile Parents-a Cohort Study.” *Fertility and Sterility* 106 (5): 1033. <http://www.elsevier.com/locate/fertnstert>.

#### Finish cohort(s)

Rantakallio, P, and A Myhrman. 1980. “The Child and Family Eight Years after Undesired Conception. The Child and Family after Undesired Conception.” *Scandinavian Journal of Social Medicine* 8 (3): 81–87. https://pubmed.ncbi.nlm.nih.gov/7209459/.

#### Indian cohort(s)

A.K., Upadhyay, Singh A., and Kumar K. 2017. “The Association Between Unintended Births and Poor Child Development in India: Evidence from a Longitudinal Study.” *Studies in Family Planning* 48 (1): 55–71.

#### Swedish cohort(s)

Höök, Kerstin. 1971. “The Unwanted Child: Effects on Mothers and Children of Refused Applications for Abortion.” In *Society, Stress, and Disease, Vol. I. The Psychosocial Environment and Psychosomatic Diseases.*, edited by Lennart Levi, 187–92. Oxford University Press.

H., Kind. 1979. “Unwanted pregnancy. Life conditions of children born after refusal of therapeutic abortion on psychiartric grounds (author’s transl).” *Schweizerische Rundschau fur Medizin Praxis = Revue suisse de medecine Praxis*, Unerwunschte Schwangerschaft. Das Schicksal der Kinder, nachdem ein Abbruch vom Psychiater gemass Art. 120 StGB abgelehnt worden ist, 68 (2): 50–55.

Blomberg, S. 1980. “Influence of Maternal Distress during Pregnancy on Postnatal Development.” *Acta Psychiatrica Scandinavica* 62 (5): 405–17.

Forssman, H, and I Thuwe. 1981. “Continued Follow-up Study of 120 Persons Born after Refusal of Application for Therapeutic Abortion.” *Acta Psychiatrica Scandinavica* 64 (2): 142–49.

## Wrong exposure

### Low weight at birth

Johnson, Rucker C, and Robert F Schoeni. 2011. “The Influence of Early-Life Events on Human Capital, Health Status, and Labor Market Outcomes Over the Life Course.” *The B.E. Journal of Economic Analysis & Policy* 11 (3).

### Reproductive Health Index

R., Prakash, Singh A., Pathak P.K., and Parasuraman S. 2011. “Early Marriage, Poor Reproductive Health Status of Mother and Child Well-Being in India.” *Journal of Family Planning and Reproductive Health Care* 37 (3): 136–45.

## Wrong outcome

### Physical development

Blomberg, S. 1980. “Influence of Maternal Distress during Pregnancy on Fetal Development and Mortality.” *Acta Psychiatrica Scandinavica* 62 (4): 298–314. https://doi.org/10.1111/j.1600-0447.1980.tb00617.x.

### Child abuse

Sidebotham, Peter, Jon Heron, and ALSPAC Study Team. 2003. “Child Maltreatment in the ‘Children of the Nineties:’ The Role of the Child.” *Child Abuse & Neglect* 27 (3): 337–52.

### Psychopathology symptoms

J.L., Roffman, Sipahi E.D., Dowling K.F., Hughes D.E., Hopkinson C.E., Lee H., Eryilmaz H., et al. 2021. “Association of Adverse Prenatal Exposure Burden with Child Psychopathology in the Adolescent Brain Cognitive Development (ABCD) Study.” *PLoS ONE* 16 (4): e0250235. <https://journals.plos.org/plosone/article/file?id=10.1371/journal.pone.0250235&type=printable>.

### Feeding behaviour, maternal depression

D.O.L., E-Andjafono, Essam B.I., Mankubu A.N., Omba A.N., and Mbuyi T.K. 2020. “Maternal affectivity during pregnancy, motherchild relationship, infant’s health and development in Kinshasa.” *Pan African Medical Journal*, Affects de la mere pendant la grossesse, relation mere-bebe, sante et developpement du nourrisson a Kinshasa, 36: 1–15. <https://www.panafrican-med-journal.com/content/article/36/203/pdf/203.pdf>.

### Cognitive and emotional parental resources available to children

Barber, Jennifer S, and Patricia L East. 2009. “Home and Parenting Resources Available to Siblings Depending on Their Birth Intention Status.” *Child Development* 80 (3): 921–39.

### Maternal wellbeing

A.M., Claridge, and Chaviano C.L. 2013. “Consideration of Abortion in Pregnancy: Demographic Characteristics, Mental Health, and Protective Factors.” *Women and Health* 53 (8): 777–94.

## Wrong control

Foster, Diana Greene, Sarah E. Raifman, Jessica D. Gipson, Corinne H. Rocca, and M. Antonia Biggs. 2019. “Effects of Carrying an Unwanted Pregnancy to Term on Women’s Existing Children.” *Journal of Pediatrics* 205: 183-189.e1. <https://doi.org/10.1016/j.jpeds.2018.09.026>.

D.G., Foster, Biggs M.A., Raifman S., Gipson J., Kimport K., and Rocca C.H. 2018. “Comparison of Health, Development, Maternal Bonding, and Poverty among Children Born after Denial of Abortion vs after Pregnancies Subsequent to an Abortion.” *JAMA Pediatrics* 172 (11): 1053–60. <http://archpedi.jamanetwork.com/issues.aspx>.

## Wrong study type

### Descriptive study

M., Blazek, Kazmierczak M., and Kielbratowska B. 2010. “An analysis of psychosocial functioning of minor mothers.” *Ginekologia i Poloznictwo*, Analiza funkcjonowania psychospolecznego niepelnoletnich matek, 18 (4): 79–90. http://www.medicalproject.com.pl/download.php?idArt=434.

P.J., Thompson, Powell M.J., Patterson R.J., and Ellerbee S.M. 1995. “Adolescent Parenting: Outcomes and Maternal Perceptions.” *Journal of Obstetric, Gynecologic, and Neonatal Nursing : JOGNN / NAACOG* 24 (8): 713–18.

Guerreiro, T B F, L I C Cavalcante, E F Costa, and M D R Valente. 2016. “Psychomotor Development Screening of Children from Kindergarten Units of Belém.” *Journal of Human Growth and Development* 26 (2): 181–89. https://doi.org/10.7322/jhgd.119262.

### Predictive model

M., Laucht, Esser G., and Schmidt M.H. 1998. “Risk and protective factors in early child development: Empirical results.” *Zeitschrift fur Kinder- und Jugendpsychiatrie und Psychotherapie*, Risiko- und schutzfaktoren der fruhkindlichen entwicklung: Empirische befunde, 26 (1): 6–20.

Usta, M.B., and K Karabekiroglu. 2020. “Does the Psychopathology of the Parents Predict the Developmental-Emotional Problems of the Toddlers?” *Noropsikiyatri Arsivi*, Ebeveynlerin psikopatolojisi, kucuk cocuklarin gelisimsel-duygusal problemlerini ongoruyor mu?, 57 (4): 265–69. https://doi.org/https://dx.doi.org/10.29399/npa.25074.

## Wrong publication type

### Background article

Eisenberg, L. 1981. “Social Context of Child Development.” *Pediatrics* 68 (5): 705–12.

### Conference abstract (all published versions were screened)

D.G., Foster, Biggs A., Raifman S., Gipson J., Kimport K., and Rocca C. 2017. “Health, Development and Maternal Bonding among Children Born Following Abortion Denial and Children Born to Women in the 5 Years after They Receive an Abortion.” *Contraception*, 2017 North American Forum on Family Planning. Atlanta, GA United States., 96 (4): 263.

D.G., Foster. 2014. “Effect of an Unwanted Pregnancy Carried to Term on Existing Children’s Health, Development and Care.” *Contraception*, ["2014 North American Forum on Family Planning. Miami, FL United States.", “(var.pagings).”], 90 (3): 305.

J., Roffman, Sipahi E., Dowling K., Hughes D., Hopkinson C., and Eryilmaz H. 2019. “Association of Adverse Prenatal Exposures with Psychopathology at Age 9-10 in the Adolescent Brain Cognitive Development (ABCD) Study.” *Neuropsychopharmacology*, 58th Annual Meeting of the American College of Neuropsychopharmacology, ACNP 2019. Orlando, FL United States., 44: 411–12.
