# Supplementary material for: Association between pregnancy planning or intention and early child development: A systematic scoping review
Source: PLOS Glob Public Health. 2023 Dec 5;3(12):e0002636. doi: 10.1371/journal.pgph.0002636 (PMC10697520; doi:10.1371/journal.pgph.0002636)
Supplement: Attachment — Submitted filename: Response_to_peer_review.docx [file pgph.0002636.s004.docx]

Top of Form

September 28, 2023

# Dr. Colleen M. Davison

Academic Editor, PLOS Global Public Health

Dear Dr. Davison,

We are grateful for the editors and reviewers careful review of our manuscript, **“Association between pregnancy planning or intention and early child development: a systematic scoping review”** (reference number PGPH-D-23-00602). Overall, we are happy that they viewed the paper positively and saw it as an important contribution to the field of global health.

Some very insightful comments were raised as directions for strengthening our manuscript. We have carefully considered these comments and have edited the paper accordingly. Below is a summary of each point for revision raised by the reviewers and our responses. We have also attached a revised manuscript with track changes. We believe the paper has been significantly strengthened because of these edits.

We look forward to your further review of our work.

With best regards,

Jorge Andrés Delgado-Ron, on behalf of the author team.

**REVIEWERS' COMMENTS:**

**Reviewer #1:**
*This systematic scoping review presents a unique approach to considering reproductive rights from the perspective of pregnancy intention and impacts on child development. The review outlines a comprehensive search and detailed strategy.*

***Point 1.*** *Overall, there is a lack of discussion regarding the interpretation of pregnancy intention. [The research] assumes that intention is a constant across the pregnancy and child life course – once unwanted, always unwanted. Not only could there be errors or misinterpretations regarding the meaning behind intention for both researcher and participant, but intention could also be a dynamic process – perspectives regarding pregnancy/parenting could shift over time, throughout the course of the pregnancy etc. See Santelli et al., 2003;^[1]^ Santelli et al., 2009;^[2]^ Borrero et al., 2014^[3]^ for more on this.*

We understand that the measurement of pregnancy intention—and its use—has been criticized in the past. The references provided by the reviewer point to high in-person variation over time. However, these same references recognize that the measure is stable at the population-level and valid for effect estimation. Santelli and colleagues (2003) write: “measures of unintended pregnancy seem reasonable, reliable and predictive at a population level.” Santelli et al. (2009) reiterate: “Longitudinal surveys demonstrate that pregnancy intentions may change over time and after childbirth; this change is not so severe, however, as to invalidate the method of retrospective reporting.” The third reference expands on the issues we can find at the person level. However, these are less relevant to population-level measures. Therefore, we added the following in the introduction:

| Original text | Revised version |
| --- | --- |
| Such effects are relevant to a woman’s health and affect children of unwanted pregnancies. | Such effects are relevant to a woman’s health and may influence children born from pregnancies that were initially perceived as unwanted. **Although past research has shown that feelings about past pregnancies can change over time and after pregnancy, population-level estimates remain consistent and retrospective reporting is considered a valid tool to assess pregnancy intention** (8,18) . |

***Point 2.*** *Overall, there is a lack of discussion regarding the … nuanced experiences and* perspectives of women *who have unwanted/unintended/mistimed pregnancies. This relates to concerns around the discussion in the Significance of Results section and interpretation of the review results as outlined below. … Attributing poorer early child development to “not being loved enough” overlooks the context – that women may not want their pregnancy at the time for a whole range of different reasons, and are situated within a range of different social/physical environments, that could impact the child’s development (some of which are briefly touched on in confounders but certainly not all). The reasons regarding pregnancy intention or not wanting a pregnancy could range from personal preference, personal capacity, social environment, inequities etc. and that these reasons all function differently in terms of pregnancy and parenting behaviours. See Auerbach et al., 2023^[4]^ as an example related to this. Re-writing the introduction and discussion sections to better contextualize women’s experiences and perspectives and considerations of the interpretation of intention would help to address some of the concerns related to the interpretation and significance of the systematized review results.*

We also added some relevant information to the interpretation as targeted interventions could be more challenging given the lack of reliability of pregnancy intention at the individual level:

| Original text | Revised version |
| --- | --- |
| After conception, assistance throughout pregnancy and strategies that target children born from unintended pregnancies and their families are also required, although it is unclear whether home-based and other effective strategies (48,49) would work when pregnancies are unwanted. | Post-conception strategies are more challenging; **pregnancy intention reports are not as reliable at the individual level as they are at the population level. The reasons behind unintended pregnancies, such as personal preferences, financial stability, emotional readiness, socio-economic challenges, and broader social inequities within a specific context, can significantly impact early child development (48). The effectiveness of strategies aimed at promoting early child development, including home-based approaches (49,50), may vary based on these characteristics Tailored support may need to be flexible and adaptive to address changing sentiments during and after pregnancy**. |
| Our findings reinforce the notion that “child development starts at conception” (Daelmans et al., 2017) but challenge health and policy systems to address even earlier reproductive life stages. In other words, ensuring that all girls and boys have access to quality early childhood development might entail creating supportive environments where women see conception as a desirable life event and to allow women who do not to access safe abortion. | Our findings reinforce the notion that “child development starts at conception” (54). Ensuring adequate development during early childhood, as mandated by target 2 on SDG 4 necessitates more than ECD investment. It requires supportive environments for women that **respect and acknowledge the complexities of their reproductive choices and emotions**; ensuring they have support and access to safe reproductive health options, including abortion, **irrespective of their initial feelings about conception** (48) |

***Point 3.*** *Overall, there is a lack … engagement with the sociopolitical contexts… Although the authors looked at studies from six countries: New Zealand (NZ), Germany, Canada, Brazil, the United States, and the United Kingdom, they don’t contextualize the information within various sociopolitical contexts. This has potentially important implications because the study is not just talking about women who may have wanted an abortion but also women who are parenting children born of those unintended/mistimed pregnancies. There are certain populations within these countries that may be more affected by lack of access to birth control, abortion services etc. and more likely to have higher rates of unwanted/unintended/mistimed pregnancies such as underserved and racialized populations in the United States and Canada. Given that it is largely structurally marginalized women that have difficulty accessing abortion services/reproductive services within these countries etc., a focus on “love” in the discussion may potentially reinforce stereotypes of “bad motherhood” related to marginalized women who may not have planned for their pregnancies but are parenting those children now.*

Thank you so much for bringing this up. We have added this to the discussion as we consider it extremely relevant:

| Original text | Revised version |
| --- | --- |
| The existing evidence is limited to developed countries and Brazil (an upper-middle income economy), making it hard to translate their findings to populations in developing countries given varying levels of investment in social services and familial and socio-cultural contexts (36,37). For context, 92% of children are born in developing countries (38). | The existing evidence is limited to developed countries and Brazil (an upper-middle income economy), making it hard to translate their findings to populations in developing countries given varying levels of investment in social services and familial and socio-cultural contexts (36,37). For context, 92% of children are born in developing countries (38). **However, lessons are relevant as some of these countries were undergoing sociopolitical transformations when the studies were conducted. In the 1960s and 70s, Germany was navigating the path towards liberalizing abortion laws, which only became more permissive in 1976. Similarly, abortion was only partially decriminalized in Canada in 1969, and access to services remained inconsistent across provinces. The Christchurch study coincided with the nation's Contraception, Sterilisation, and Abortion Act in 1977, implying restricted access to abortion services at that time. Finally, while ‘Roe v. Wade’ significantly increased women's access to abortion in the US, starting in 1973, disparities persisted among marginalized communities that also had historical reports of higher levels of unintended pregnancies (i.e., Hispanics and African Americans) (39).** |

***Point 4****. Lines 330-334 under significance of results^[5]^ seem to be implying that poorer early child development stems from women/families/circumstances in which children are “not loved enough”. This seems like a large assumption that overlooks many different contexts and experiences of women that seems problematic for several reasons. [The statement] assumes pregnancy intention and love are the same or directly related. Women may not have wanted or planned for these pregnancies because they knew they were unable to provide for the child due to a variety of circumstances related to capacity and social inequities rather than lack of love. In fact, arguably not wanting a pregnancy could be an expression of love – love for oneself, love for one’s existing family, even love for the idea of an unborn child if they believe carrying and birthing that child would deliver them into harm/oppression etc. See Manze et al., 2021;^[6]^ Leath et al., 2022^[7]^ as examples related to this.*

Thanks for pointing this out. While we originally aimed to allow unwanted children to express their views, we understand how this might create all kinds of issues. We decided to delete this sentence.

| Original text | Revised version |
| --- | --- |
| ~~When the WHO–UNICEF–Lancet Commission asked children from around the world what made them ‘happy or healthy,’ a boy from Ibadan, Nigeria, said it was really important “that my parents love me and that we are complete” (Clark et al., 2020). Our findings echo his message:~~ children born from unintended or unplanned pregnancy seem to have poorer development during early childhood. | Children born from unintended or unplanned pregnancy seem to have poorer development during early childhood |

***Point 5****. The proposed interventions/actions^[8]^ in lines 342-347 are unclear in terms of specifics and seem to focus on children born into families of unintended pregnancies. Given that certain populations are more likely to have unwanted/unintended/mistimed pregnancies AND are more likely to face social inequities, racism/discrimination etc. within these countries it would be helpful to provide more details as the recommendations potentially targets the women and children in a way that also seems to ignore the dynamic nature of pregnancy/parenting intention and the socio-political context. For instance, flagging women who have unintended pregnancies as requiring intervention could lead to harmful practices, especially for marginalized women, who face discrimination, birth alert practices, inequitable child welfare practices etc.*

We addressed this by adding:

**Birth alerts, though intended as protective measures, have often had the unintended consequence of perpetuating systemic discrimination, particularly against Indigenous mothers in Canada. Such practices, especially when intersecting with issues like unintended pregnancies, can exacerbate the already profound social inequities and challenges these marginalized populations face.**

***Point 6.*** *While I agree with the authors that there is a potential need to create supportive environments where women who do not desire pregnancy can access abortion, and that conception should be a desired life event, and can understand the limitations around discussing many complex concepts, overall the discussion and interpretation of results seems to both erase women (their knowledge/experiences/positions) in their lives AND yet almost villainizes them within the context of child development. Lines 324-326 state^[9]^ the authors overlooked other results that tried to offer a more nuanced view of the relationship. It’s unclear the rationale behind this – was it for ease of analysis? How does this relate overall to the interpretation and understanding of the results?*

We appreciate the thoughtful feedback provided. In our revised manuscript, we have made a concerted effort to address the concerns raised, particularly in terms of contextualizing women’s experiences and perspectives, as shown above. Our intention is to highlight the complex interplay of factors surrounding unintended pregnancies, and we believe this complexity is now more thoroughly represented.

We also edited the mentioned lines to clarify what we meant:

| Original text | Revised version |
| --- | --- |
| Moreover, we overlooked other results that tried to offer a more nuanced view of the relationship between pregnancy intention/planning and child development (i.e., categories of variables groups in nested models). We provided a narrative synthesis and a model compilation so that readers can more easily access the existing literature for more in-depth analyses. | Moreover, **we did not analyze mediation or moderation results** that tried to offer a more nuanced view of the relationship between pregnancy intention/planning and child development (i.e., categories of variables groups in nested models) **because it was outside the scope of the project. It is imperative to recognize the nuances and complexities in the relationship between pregnancy intention/planning and child development, which is why** we provided a narrative synthesis and a model compilation so that readers can more easily access the existing literature for more in-depth **understanding of the interplay between these factors at the population-level.** |

***Point 7.***

*Lines 339-342^[10]^ reference a goal of lifelong learning. It’s unclear what goal/objective this sentence is referring to – is it presumed the woman’s goal of lifelong learning?*

| Original text | Revised version |
| --- | --- |
| Sexual education for both genders, adequate access to the full range of contraception methods (including “morning-after” pills), reproductive life plan development, and access to abortion services (46) might be paramount to achieving the goal of ensuring lifelong learning. | Safeguarding and expanding reproductive rights are key aspects of improving health and social outcomes for women. Our results suggest that protecting these rights could have further positive impacts on other developmental goals. Comprehensive sexual education for both genders, along with unfettered access to a spectrum of contraception methods—including “morning-after” pills—and abortion services (Johnson-Mallard et al., 2017), **are essential not only for women's autonomy but for the subsequent well-being and development of their children. Individuals empowered with knowledge and the means to control their reproductive choices are better positioned to provide for their children's needs, thereby influencing their educational opportunities and outcomes. Our review suggests that pre-conception strategies could contribute directly to the objective of ensuring stronger educational outcomes**. |

1. Santelli, J., Rochat, R., Hatfield-Timajchy, K., Gilbert, B. C., Curtis, K., Cabral, R., ... & Unintended Pregnancy Working Group. (2003). The measurement and meaning of unintended pregnancy. Perspectives on sexual and reproductive health, 94-101.
2. Santelli, J. S., Lindberg, L. D., Orr, M. G., Finer, L. B., & Speizer, I. (2009). Toward a multidimensional measure of pregnancy intentions: evidence from the United States. Studies in family planning, 40(2), 87-100.
3. Borrero, S., Nikolajski, C., Steinberg, J. R., Freedman, L., Akers, A. Y., Ibrahim, S., & Schwarz, E. B. (2015). “It just happens”: a qualitative study exploring low-income women’s perspectives on pregnancy intention and planning. Contraception, 91(2), 150-156.
4. Auerbach, S. L., Coleman‐Minahan, K., Alspaugh, A., Aztlan, E. A., Stern, L., & Simmonds, K. (2023). Critiquing the Unintended Pregnancy Framework. Journal of Midwifery & Women's Health, 68(2), 170-178.
5. «When the WHO–UNICEF–Lancet Commission asked children from around the world what made them ‘happy or healthy,’ a boy from Ibadan, Nigeria, said it was really important “that my parents love me and that we are complete” (Clark et al., 2020). Our findings echo his message: children born from unintended or unplanned pregnancy seem to have poorer development during early childhood. »
6. Manze, M. G., Romero, D. R., De, P., Hartnett, J., & Roberts, L. (2021). The association of pregnancy control, emotions, and beliefs with pregnancy desires: A new perspective on pregnancy intentions. PloS one, 16(3), e0249032.
7. Leath, S., Wright, P., Charity-Parker, B., & Stephens, E. (2022). Exploring Black women’s pathways to motherhood within a reproductive justice framework. Qualitative Health Research, 32(4), 694-709.
8. «After conception, assistance throughout pregnancy and strategies that target children born from unintended pregnancies and their families are also required, although it is unclear whether home-based and other effective strategies (Jeong et al., 2021; Kumar et al., 2022) would work when pregnancies are unwanted. Finally, women who have experienced multiple unintended pregnancies could also be targeted by other policy efforts, given that other interventions might be less effective on them (e.g., women forced to conceive). »
9. «Moreover, we overlooked other results that tried to offer a more nuanced view of the relationship between pregnancy intention/planning and child development (i.e., categories of variables groups in nested models). »
10. «Sexual education for both genders, adequate access to the full range of contraception methods (including “morning-after” pills), reproductive life plan development, and access to abortion services (Johnson-Mallard et al., 2017) might be paramount to achieving the goal of ensuring lifelong learning. »
